# Acromegaly

**DOI:** 10.1186/1750-1172-3-17

**Published:** 2008-06-25

**Authors:** Philippe Chanson, Sylvie Salenave

**Affiliations:** 1 Assistance Publique-Hôpitaux de Paris, Hôpital de Bicêtre, Service d'Endocrinologie et des Maladies de la Reproduction and Centre de Référence des Maladies Endocriniennes Rares de la Croissance, Paris, France; 2 Université Paris-Sud 11, Paris, France; 3 INSERM U693, Paris, France

## Abstract

Acromegaly is an acquired disorder related to excessive production of growth hormone (GH) and characterized by progressive somatic disfigurement (mainly involving the face and extremities) and systemic manifestations. The prevalence is estimated at 1:140,000–250,000. It is most often diagnosed in middle-aged adults (average age 40 years, men and women equally affected). Due to insidious onset and slow progression, acromegaly is often diagnosed four to more than ten years after its onset. The main clinical features are broadened extremities (hands and feet), widened thickened and stubby fingers, and thickened soft tissue. The facial aspect is characteristic and includes a widened and thickened nose, prominent cheekbones, forehead bulges, thick lips and marked facial lines. The forehead and overlying skin is thickened, sometimes leading to frontal bossing. There is a tendency towards mandibular overgrowth with prognathism, maxillary widening, tooth separation and jaw malocclusion. The disease also has rheumatologic, cardiovascular, respiratory and metabolic consequences which determine its prognosis. In the majority of cases, acromegaly is related to a pituitary adenoma, either purely GH-secreting (60%) or mixed. In very rare cases, acromegaly is due to ectopic secretion of growth-hormone-releasing hormone (GHRH) responsible for pituitary hyperplasia. The clinical diagnosis is confirmed biochemically by an increased serum GH concentration following an oral glucose tolerance test (OGTT) and by detection of increased levels of insulin-like growth factor-I (IGF-I). Assessment of tumor volume and extension is based on imaging studies. Echocardiography and sleep apnea testing are used to determine the clinical impact of acromegaly. Treatment is aimed at correcting (or preventing) tumor compression by excising the disease-causing lesion, and at reducing GH and IGF-I levels to normal values. Transsphenoidal surgery is often the first-line treatment. When surgery fails to correct GH/IGF-I hypersecretion, medical treatment with somatostatin analogs and/or radiotherapy can be used. The GH antagonist (pegvisomant) is used in patients that are resistant to somatostatin analogs. Adequate hormonal disease control is achieved in most cases, allowing a life expectancy similar to that of the general population. However, even if patients are cured or well-controlled, sequelae (joint pain, deformities and altered quality of life) often remain.

## Disease name and synonyms

**Acromegaly **(derived from the Greek words "*akros*", extremities, and "*megas*", big). This term was proposed by Pierre Marie, a famous French neurologist working in La Salpetrière Hospital, in Paris, who published the first description of the disease and its pathology in 1886. It is used when the disease begins in adulthood.

**Gigantism: **when the disease begins during childhood.

**Prosopectasia **(derived from the Greek words "*prosopon*", face, and "*ektasis*", stretching): used by Verga, an Italian anatomist, in 1864.

## Definition

Acromegaly is characterized by an acquired progressive somatic disfigurement, mainly involving the face and extremities, but also many other organs, that is associated with systemic manifestations. The disease is related to the excessive production of growth hormone (GH). This GH hypersecretion originates from a monoclonal benign pituitary tumor (adenoma) in more than 90% of cases.

## Epidemiology

Acromegaly is a rare disease, with a prevalence of 40 to 70 cases per million inhabitants and an annual incidence of 3 to 4 new cases per million inhabitants [1]. However, a recent study performed in Belgium suggests that pituitary adenomas may be more prevalent than previously thought, and thus the prevalence of acromegaly would be around 100–130 cases per million inhabitants [2]. A very recent epidemiological study (conducted in Germany [3] where screening of acromegaly was performed by means of systematic insulin-like growth factor-I (IGF-I) measurement in primary care patients of the general population on a given day) found a prevalence of biochemical acromegaly even higher (1,043 per million). Such high figures need to be confirmed. Owing to its insidious onset, acromegaly is often diagnosed late (4 to more than 10 years after onset), at an average age of about 40 years. The disease affects both men and women equally [4-6].

## Clinical description [6-9]

### The dysmorphic syndrome [6,10]

**Figure 1:**
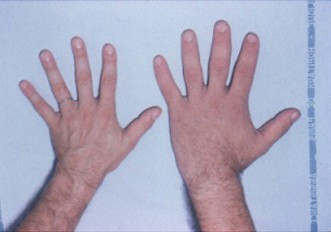
As compared with the hand of a normal person (left), the hand of a patient with acromegaly (right) is enlarged, the fingers are widened, thickened and stubby, and the soft tissue is thickened.

**Figure 2:**
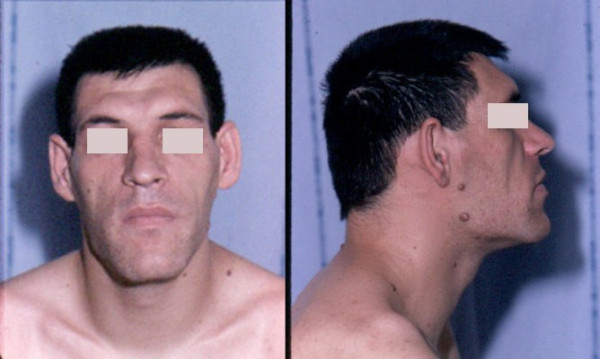
**Facial aspect of a patient with acromegaly**. The nose is widened and thickened, the cheekbones are obvious, the forehead bulges, the lips are thick and the facial lines are marked. The forehead and overlying skin is thickened, sometimes leading to frontal bossing.

The extremities (hands and feet) are broadened, the fingers are widened, thickened and stubby, and the soft tissue is thickened (Figure 1). The patient may have had to enlarge his or her ring in recent years, or to change shoe size. The facial aspect is characteristic, and patients with established acromegaly are generally alike in this respect: the nose is widened and thickened, the cheekbones are obvious, the forehead bulges, the lips are thick and the facial lines are marked (Figure 2). The forehead and overlying skin is thickened, sometimes leading to frontal bossing. There is a tendency towards mandibular overgrowth with prognathism, maxillary widening, teeth separation and jaw malocclusion. Photographs show a slow, insidious transformation over several years. The diagnosis is often raised by a doctor who has never seen the patient before. The deformations can also affect the rest of the skeleton and, in severe chronic forms, dorsal kyphosis with deformation of rib cage may be observed, leading to the classical "*punchinello*" aspect, especially when GH hypersecretion begins prior to closure of the epiphyses.

### Symptoms

Acromegaly can cause a variety of symptoms, such as malodorous sweating (especially at night); headache (whether the pituitary adenoma is large or small); acroparesthesia (carpal tunnel syndrome); and joint pain. A progressive deepening of the voice is also observed.

### Skin changes

Nearly 70% of patients have sweaty, oily skin. Skin thickening is due to glycosaminoglycan deposition and to increased collagen production by connective tissue. Skin tags are frequent and may be a marker of colonic polyps. Raynaud's disease is present in one-third of cases.

### Bone changes

#### Craniofacial

**Figure 3:**
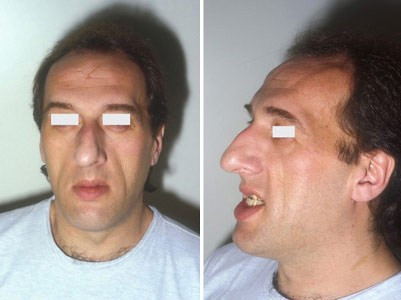
Mandibular overgrowth leads to prognathism, maxillary widening, teeth separation and jaw malocclusion.

In response to both GH and IGF-I, periosteal new bone formation leads to an increase in skeletal growth, especially at the level of the mandible (prognathism); jaw thickening, teeth separation, frontal bossing, malocclusion, and nasal bone hypertrophy are the usual facial bony deformities seen in acromegaly (Figure 3).

Radiography shows a thickening of the cranial vault and protuberances, frontal internal hyperostosis, condensation of the walls of the sella turcica with clinoid hypertrophy. Hypertrophy of the sinuses, especially the frontal sinuses, is also clearly visible. This, along with laryngeal hypertrophy, explains why the voice in acromegaly tends to become deeper and has a sonorous resonance.

#### Extremities

These changes are not only due to soft tissue hypertrophy and excess growth of bone and cartilage but also to bone deformation. Indeed, radiography is abnormal in half of the cases, showing distal tufting of the phalanges, widening of the base of phalanges with osteophyte formation, enthesopathy (mineralization of ligamentous insertion), widening of diaphysis in cortical bone, and widening of joint spaces due to cartilage hypertrophy [11].

#### Trunk

**Figure 4:**
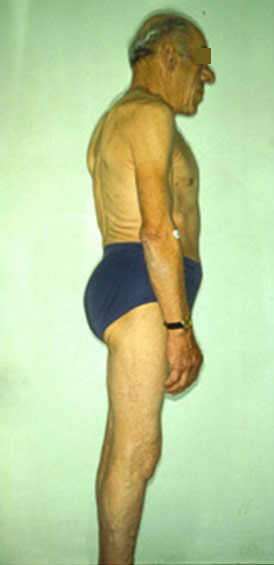
**In longstanding forms, bony deformation affects the spine, with upper dorsal kyphosis and compensatory lumbar hyperlordosis**. The thorax is deformed due to protuberance of the lower portion of the sternum, and by elongation and divergence of the ribs.

Bony deformation also affects the spine, with upper dorsal kyphosis and compensatory lumbar hyperlordosis. Vertebral enlargement, widened intervertebral spaces and osteophyte formation also being observed. The thorax is deformed due to protuberance of the lower portion of the sternum, and by elongation and divergence of the ribs (due to overgrowth of the chondrocostal joints) (Figure 4).

#### Limbs

Imaging studies show diaphyseal cortical thickening of the long bones and widened joint spaces, sometimes with osteophytes.

#### Bone mineral density

Bone remodeling is stimulated in acromegaly. Cortical bone thickens (as measured by the metacarpal index and histomorphometric parameters) and its porosity is diminished. The trabecular bone mass may be decreased, normal or increased. Measurement of spinal bone mass can give contradictory results, probably because acromegaly is often associated with other endocrine disorders that interfere with bone mass. In general, bone mass is normal in the lumbar spine in patients with isolated acromegaly, but is decreased in patients with associated hypogonadism [12], as it is generally the case for hypogonadism whatever its cause. Vertebral compression is rare and is usually due to other causes.

### Rheumatologic complications

#### Peripheral arthropathy

Peripheral joint symptoms are very frequent [13,14]. Arthralgia and myalgia occur in 30%–70% of patients. All the joints can be affected (typically the large joints: knees, shoulders, hands, wrists and hips). Acromegalic arthropathy develops within an average of ten years after diagnosis. The arthralgia is mainly mechanical, degenerative, non-inflammatory in origin but features of osteoarthritis may develop in some patients. Joint mobility (especially of the shoulders) can be limited in the later stages of the disease. Joint effusion is rare and synovial aspirate shows a generally degenerative picture without evidence of inflammation, but may also point to the presence of calcium microcrystals (associated chrondrocalcinosis).

Physical examination of joints often provides little information. The abnormalities are generally minor as compared to the subjective functional discomfort. The shoulders and hips may show a loss of mobility and function. In contrast, some patients have joint hyperlaxity. There is no correlation between the presence (or severity) of arthropathy and the age of onset of acromegaly, or the mean GH or IGF-I concentration at baseline or during follow-up. Arthropathy appears to be more frequent after age of 45 years.

Radiological studies show a widening of the joint spaces, reflecting hypertrophy of the hyaline cartilage, the presence of osteophytes, bone proliferation at the attachment sites of tendons and ligaments, periarticular calcium deposit and exostosis of the bone surface. The joint space subsequently diminishes due to destructive arthropathy. Sonography shows a thickening of the cartilage in the shoulder, wrist and knee joints, which improves during treatment for acromegaly [15].

The arthropathy progresses inexorably in advanced stages and unpredictably in minor forms. It is not influenced by successful treatment of acromegaly, with the exception of diffuse articular symptoms and some sites of pain.

#### Spinal involvement

The estimated prevalence of spinal involvement is about 40% to 50%. Backache is more frequent at the level of the lumbar spine than cervical or dorsal spine. The pain is mainly mechanical in nature, but inflammatory features can occur (16%). Spinal involvement may be accompanied by nerve compression. Occasionally, bilateral intermittent claudication reveals lumbar spinal stenosis

Radiological examination shows typical features: ossification of the anterior and lateral surface of the vertebral bodies contributing to enlarging their anteroposterior diameter; a biconcave vertebral appearance and scalloping of the vertebral bodies (exaggerated concavity of the posterior vertebral wall). The mechanism is poorly understood, and may involve hypertrophy of the intraspinal soft tissues (ligamentous hypertrophy, epidural lipomatosis) or of the bone. In more severe cases, the process of ossification of the anterior surface of the vertebral bodies can bridge the disc space and give the aspect of diffuse idiopathic skeletal hyperostosis.

### Neuropathies

Symptomatic carpal tunnel syndrome is frequent (occurs in 20%–50%, up to 75% of patients at diagnosis). Nerve conduction studies have documented that the vast majority of acromegalic patients have subclinical abnormalities of nerve conduction. Magnetic resonace imaging (MRI) shows an increase in the amplitude and intensity of the median nerve signal in the patients with symptomatic carpal tunnel syndrome compared to asymptomatic patients [16]. The mechanism appears to involve median nerve edema more than extrinsic compression due to an excess of connective tissue, bony or synovial hypertrophy, or an increase in extracellular fluid within the carpal tunnel itself with Schwann cell demyelination. The nerve edema improves when GH and IGF-I levels fall, suggesting that hormonal control is a key prerequisite for improving neurological status. Sometimes, however, the carpal tunnel syndrome persists.

### Cardiovascular manifestations

#### Arterial hypertension

Hypertension occurs in 20% to 50% of patients. Its prevalence increases with time after the onset of acromegaly, the GH level, and age. It is at least partly due to chronic hypervolemia (the plasma volume is 10% to 40% above normal due to increased renal sodium reabsorption at the distal tubule level) [17,18]. Hypertension can also result from endothelial dysfunction [19]. Neither renin angiotensin aldosterone nor sympathetic systems seem to be involved in the pathogenesis of hypertension. Insulin resistance and diabetes may also play a role in the onset of hypertension [20,21]. Sleep apnea syndrome is likely to contribute also to the pathogenesis of hypertension.

#### Specific cardiomyopathy

Cardiac involvement is a consistent feature of acromegaly. Many lines of evidence, especially from experimental studies, point to the existence of specific cardiac disorders in acromegaly, independently of coronary involvement (found in a minority of patients nowadays) and valve disorders [8,22,23].

Initially, the cardiac involvement is asymptomatic (at least at rest), and consists mainly of myocardial hypertrophy (of the interventricular septum and left ventricular posterior wall), as assessed by echocardiography, but the dimensions of the left ventricle are normal (concentric hypertrophy). It can occur in the absence of hypertension, and even in young patients (< 30 years), reflecting the role of GH itself on the myocardium. Hypertension further aggravates cardiac hypertrophy. Echocardiography and isotope studies show altered diastolic function (abnormal left and right ventricle filling) related to abnormal relaxation (parietal stiffness is, at least in part, probably linked to edematous infiltration of the ventricular wall and perhaps also to a certain degree of fibrosis). Clinical symptoms such as dyspnea during exercise may be observed while the patient is asymptomatic at rest. Systolic function is normal at this stage (again, at least at rest), thanks to increased myocardial contractility. A hyperkinetic syndrome (increased cardiac index) is always present. Systolic function is altered during exercise, however. Even at this early stage, arrhythmias and/or conduction disorders may occur. Their prevalence in acromegaly was underestimated for many years. In fact, ventricular premature complexes occur in about 40% of patients with acromegaly, and, in one study, systematic 24 h Holter electrocardiogram (ECG) recordings showed complex ventricular arrhythmias in 48% of patients (compared to only 12% of controls). Most of these arrhythmias are subclinical and persist despite successful treatment of acromegaly. Myocardial remodeling, hypertrophy and fibrosis all are likely to play a role in their onset.

Congestive heart failure can occur if the cardiac disorders progress (if GH hypersecretion persists and, probably, if other risk factors such as diabetes, hypertension, and sleep apnea are also present); functional signs appear on effort at first, before becoming permanent. At this stage, echocardiography shows variable degrees of cavity dilation. Fortunately, these severe forms are now far less frequent (prevalence 3%) [24].

A number of cardiovascular parameters improve during effective treatment of acromegaly, even if some lesions may appear to be irreversible in certain patients. In general, younger patients and patients with a relatively short history of acromegaly show better "recovery" (from diastolic disorders, myocardial hypertrophy or systolic dysfunction). In contrast, when dilated congestive heart failure occurs, cardiac function (especially systolic function) may show a short-term improvement, allowing some patients to survive or to avoid heart transplantation, but the longer-term prognosis is worse than that of patients with heart failure due to other causes (5-year mortality rate 37%).

#### Valve disease

The increased prevalence of valve disorders can also contribute to the onset or aggravation of the heart disease in patients with acromegaly [25]. The risk of valve disease increases with time from onset [26], and these abnormalities often persist after effective treatment of acromegaly. They may be related to fibrotic changes.

### Metabolic complications

Physiologically, GH increases blood glucose levels, exerts a lipolytic effect, and promotes triglyceride hydrolysis into free fatty acids and glycerol.

GH excess leads to insulin resistance at the level of the liver or in the periphery that leads to hyperinsulinemia. The prevalence of diabetes in acromegalic patients ranges from 20% to 56%, and that of glucose intolerance ranges from 16% to 46%, depending on the series [8]. As long as the compensatory increase in insulin secretion by pancreatic **β **cells counterbalances the reduction in insulin sensitivity, glucose tolerance remains normal. Impaired glucose tolerance occurs when insulin secretion is altered, and is followed by diabetes. There is a link between glucose tolerance, hypertension and acromegalic cardiomyopathy [20].

Acromegaly is associated with decrease in fat mass and increase in lean body mass [27].

### Respiratory complications

Sleep apnea affects 60%–80% of all patients with acromegaly (more often men) and 93% of patients with signs of this disorder. Sleep apnea is more likely to be sought in patients who snore (reported by 78% of patients with acromegaly) and those with daytime sleepiness (51%), or morning fatigue and morning headache (16%). Sleep apnea may be a contributory factor in hypertension and cardiovascular disease. In most cases the apnea is obstructive, but one-third of patients have central apnea. Obstructive apnea is linked to anatomical changes due to mandibular and maxillary growth, soft-tissue thickening (especially of the palate and uvula) and changes in the angles of the different bone segments, leading to hypercollapsibility of the posterior and lateral hypopharyngeal walls. Hypertrophy of the tongue also plays a role [28], as does hypertrophy of the submaxillary glands.

Changes in respiratory function are frequent but less well documented. Anatomical modifications of thoracic bones and cartilage (leading to profound changes in the geometry of the rib cage) and mechanical changes in thoracic elasticity and inspiratory muscles can lead to ventilatory disorders. Respiratory muscle strength is also abnormal. The inspiratory time is shorter and the breathing frequency may increase.

Patients with acromegaly often have an increase in their total lung capacity (81% of men and 56% of women), owing to an increase in alveolar volume. An obstruction is found in 20% to 30% of patients (small airway or upper airway narrowing). Subclinical hypoxemia may be present. No ventilation-perfusion mismatching has been demonstrated.

The apnea-hypopnea index improves during effective treatment of acromegaly, along with the obstructive apnea index and oximetry values [28,29]. However, while apnea can disappear in some patients whose acromegaly is cured, other require nocturnal positive end expiratory pressure for persistent sleep apnea.

### Neoplasia and acromegaly

#### Gastrointestinal tumors

The issue of colon cancer risk in acromegaly is controversial [30]. The relative risk of colon cancer, compared with the general population, has been widely overestimated at 10 to 20, whereas it is, in reality, probably only 2 to 3 [31,32]. As colon cancer may be the consequence of colon polyps degeneration, many studies have been done to assess the prevalence of colon polyps in patients with acromegaly. Prospective studies show that up to 45% of patients with acromegaly have colonic polyps, which are adenomatous in 24% of cases [33] and can arise from all sites of colon. There is no clear correlation between GH and IGF-I concentrations and the incidence of colonic polyps. Recommendations concerning colonoscopy in acromegaly are a matter of controversy. It seems reasonable to propose that, unless intestinal symptoms occur earlier, colonoscopy has only to be done first at age 50 years, whatever the progressive status and duration of acromegaly, or the history of colonic disease. As always, colonoscopy must be preceeded by careful bowel preparation and be done by a skilled operator, because it is often difficult in this setting (patients with acromegaly have a longer colon). When an adenomatous colonic polyp is discovered, an interval of three years before repeating the examination seems reasonable.

#### Thyroid nodules

Goiter is found in 25% to 90% of patients with acromegaly. The risk of developing thyroid nodules increases with the time since the onset of acromegaly. Multinodular goiter is autonomous in 10% to 20% of patients, sometimes causing patent thyrotoxicosis. Thyroid nodules are generally harmless, and the risk of thyroid cancer does not seem to be higher than in the general population.

*Other cancers *(lung, breast, prostate, *etc*.) are not over-represented in patients with acromegaly [30].

## Etiology [34,35]

### Acromegaly of pituitary origin

More than 95% of patients with acromegaly have a benign monoclonal pituitary adenoma which develops from the somatotrope cells that normally produce GH in the pituitary. Thus, these adenomas are termed somatotrope adenoma.

#### a) Somatotrope pituitary adenomas

Somatotrope pituitary adenomas may be pure or mixed. Pure somatotrope pituitary adenomas (60%) contain either cells rich in secretory granules that show diffuse immunostaining (such densely granulated somatotrope adenoma are observed in older patients with slow disease progression) or cells poor in secretory granules with scattered immunolabelling (sparsely granulated somatotrope adenomas are seen in younger patients with a more rapidly progressing disease) [34]. Some of these pure somatotrope adenomas also express free alpha-subunit, which is common to the glycoprotein hormones follicle-stimulating hormone (FSH), luteinizing hormone (LH), thyroid-stimulating hormone (TSH) and chorionic gonadotropin hormone (CG, co-localized in the same cells or even in the same granules as GH) [36].

Some adenomas are mixed. Mixed GH- and prolactin (PRL)-secreting adenomas are frequent (25%). Some adenomas contain both cell types, while other develop from a mammosomatotropic stem cell and consist of more mature monomorphic cells that express both GH and PRL [34]. Rare adenomas can also co-secrete an excess of TSH, in which case the clinical picture combines acromegaly and hyperthyroidism with inappropriate secretion of TSH [37,38]. Very rarely, corticotrophin (ACTH) hypersecretion may also be found.

#### b) GH-secreting carcinomas

In the large majority of cases adenomas are benign. Exceptional pituitary carcinomas (less than 20 cases published) may be observed and the presence of distant metastases is generally required to support the diagnosis of malignancy [39].

#### c) Silent somatotrope adenomas

Some somatotrope adenomas are not associated with systemic GH hypersecretion or GH hypersecretion is very mild. In such cases, clinical acromegaly is not observed, but GH immunolabeling of the excised tumor (generally resected because of local tumor symptoms) is positive [40]. However, when analyzed very carefully at hormonal level, it may be possible to discover subtle abnormalities in GH/IGF-I hypersecretion in these cases [41].

#### d) Pathogenesis of somatotrope adenomas

The pituitary/hypothalamic origin of these adenomas is controversial [42]. Some lines of evidence point to a hypothalamic origin. In this case, the main actor would be growth-hormone-releasing hormone (GHRH), which can cause not only hyperplasia of somatotrope cells but also, as demonstrated in some animal models, actual adenomas. In contrast, the monoclonal nature of the tumors and the absence of relapse after total tumor resection points instead to a pituitary origin [6]. In fact, the initiation and/or progression of malignant transformation of normal somatotropes could be due to a polyclonal hyperplastic response of these cells secondary to hypothalamic dysregulation. The prerequisite for an abnormal response to pathological GHRH secretion may be the existence of a mutation in the somatotrope cell. Most human somatotrope adenomas seem to be associated with clonal expansion [43] of cells bearing a somatic mutation. However, as for the other types of pituitary adenomas, isolation of a single causative factor in sporadic pituitary tumorigenesis has proved difficult. A mutated Gs**α **protein has been identified in up to 40% of somatotrope adenomas. Mutations at two critical sites (gsp mutations) inhibit GTPase activity and lead to constitutive adenyl-cyclase activation [44]. In the pituitary, loss of heterozygosity on chromosomes 11, 13 and 9 (particularly in invasive macroadenomas), and an activating gene (*PTTG*, pituitary tumor transforming gene), also play a role. This latter gene (a securin homolog) is over-expressed in functional pituitary tumors, which could lead to aneuploidy [45]; the degree of over-expression correlates with tumor size and invasiveness.

Finally, even if it is clear that somatotropes cells are altered in somatotrope adenomas, the sequence of events leading to their clonal expansion seems to be multifactorial. An activated oncogene may be necessary to initiate tumorigenesis, while promotion of cell growth may require GHRH or other growth factors, such as bFGF (basic fibroblast growth factor) [46,47].

#### e) Genetic syndromes with acromegaly

McCune-Albright syndrome, which is associated with multiple fibrous bone dysplasia, precocious puberty and *café-au-lait *spots, can be accompanied by acromegaly. This syndrome is related to a somatic mutation that activates the alpha subunit of Gs protein [48].

Acromegaly can also be associated with hyperparathyroidism, neuroendocrine tumors (*e.g*. gastrinoma, insulinoma or a non-functional pancreatic tumor), adrenal and other endocrine and non-endocrine tumors in patients with multiple endocrine neoplasia type 1 (MEN1), which is related to a germline mutation of the *menin *gene in many cases [49,50].

When acromegaly is associated with bilateral pigmented micronodular adrenal hyperplasia (causing ACTH-independent hypercorticism) and with cutaneous lesions or cardiac myxomas, the patient should be screened for the Carney complex, which is often related to a germline mutation of the regulatory 1-**α **subunit of protein kinase A (PRKAR1A) [51,52].

Very recently, familial acromegaly related to germline mutations of the *AIP *(aryl hydrocarbon receptor interacting protein) gene have been described [53]. These mutations may also, albeit rarely, be responsible for sporadic cases of acromegaly, in particular in young patients [54-56].

### Extrapituitary acromegaly

GH hypersecretion does not always have a pituitary origin. Acromegaly can be due to eutopic hypothalamic GHRH hypersecretion (gangliocytoma, hamartoma, choristoma, glioma, *etc*.) or, more often, to ectopic, peripheral GHRH hypersecretion (pancreatic or bronchial carcinoid tumor) that stimulate the normal somatotropes to become hyperplastic and to hypersecrete GH. The diagnosis is based on plasma GHRH assay (revealing excess secretion) and on identification of the GHRH-secreting endocrine tumor [57].

GH can also be hypersecreted by an ectopic pituitary adenoma (sphenoidal sinus, petrous temporal bone, nasopharyngeal cavity) or, in exceptional cases, by a peripheral tumor (pancreatic islet tumor or lymphoma) [58,59].

## Diagnosis of acromegaly

The diagnosis of acromegaly is clinical and needs to be confirmed biochemically. Clinical diagnosis is suggested by the typical disfigurement of the patient related to progressive acral enlargement and modification of facial appearance, as assessed by serial photographs. Diagnosis is made biochemically by the findings of increased serum GH concentrations that are not suppressed following an oral glucose load (oral glucose tolerance test, OGTT). An increase (with reference to the age-adjusted normal range) in the serum concentration of IGF-I), the main GH-dependent growth factor, confirms the diagnosis.

### GH assays

The first GH assays, which began to be used 35 years ago, were polyclonal competitive radio-immunoassays (RIAs); their sensitivity was poor. Thereafter, over the last 25 years, non-competitive two-site antibody radioimmunometric assays (IRMAs) have been introduced allowing for enhanced sensitivity. Fifteen years ago, non-isotopic two-site antibody assays began to become available, with the major practical advantage being that some were automated [60]. This last type of assays is the most frequently used today, at least in Europe. The differences in analytical methodologies (RIA, IRMA, immunochemiluminescent assay -ICMA-, Enzyme-linked immunoabsorbent assay -ELISA-) are one explanation for the variability in GH results. GH circulates in plasma as a mixture of different molecular forms: 22 kDa GH, 20 kDa GH, a GH-binding protein (GH-BP) linked form, dimers and polymers [61]. Use of polyclonal or monoclonal antibodies specific for the predominant 22 kDa or for several of these different isoforms contributes to bias.

Many standard GH preparations were used previously to calibrate GH. Manufacturers were recently advised to calibrate their GH assay kits with the international standard (IS) 98/574, which was established with recombinant GH. According to a recent European consensus statement on the standardization of GH assays [62], "the availability of the second International Standard (IS) for GH (WHO IS 98/574), a recombinant material consisting of 22 kDa GH of more than 95% purity, provides the opportunity for adoption of a single calibrant for GH immunoassays. IS 98/574's well-defined chemical and physical properties allow it to meet European Union legislation calls for all laboratory results to be traceable to a defined material (In vitro Diagnostics Medical Devices Directive, 98/79/EC)." As a first step to standardizing GH measurement", they "recommend the reporting of GH concentrations in **μ**g/l of IS 98/574 (1 **μ**g corresponding to 3 IU somatropin)".

### Which GH cutoff use for the diagnosis?

**Figure 5:**
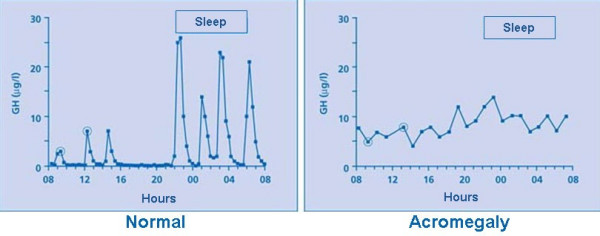
Frequent blood sampling with serum GH measurement shows that in normal subjects (left panel) GH can fluctuate between undetectable levels (most of the time) and peaks of up to 30 **μ**g/l (90 mIU/l), owing to the episodic nature of GH secretion, while in patients with acromegaly (an example is given on right panel), GH hypersecretion is continuous and GH never returns to undetectable levels.

The basal plasma GH level (in the morning for example, or at randomly selected times) is elevated in acromegaly. However, high GH concentrations can also be found in healthy subjects, owing to the episodic nature of GH secretion, that can fluctuate between undetectable levels (most of the time) and peaks of up to 30 **μ**g/l (90 mIU/l) (Figure 5). According to a 2000 Consensus statement [63], basal GH and IGF-I measurement must be done when acromegaly is suspected. A GH concentration below 0.4 **μ**g/l (1.2 mIU/l) plus a normal IGF-I level rules out acromegaly. If the GH is above 0.4 **μ**g/l (1.2 mIU/l) and/or if the IGF-I is elevated (as compared with age-adjusted normal range), an oral 75 g glucose load (oral glucose tolerance test -OGTT-) must be performed. If the lowest GH value (nadir) during OGGT is below 1 **μ**g/l (3 mIU/l), acromegaly can be ruled out. If it remains above 1 **μ**g/l (3 mIU/l), acromegaly is confirmed. As detailed below, with the generalized use of very sensitive assays nowadays, it has recently been considered that this cutoff should be decreased to 0.3 **μ**g/l (0.9 mIU/l). Paradoxically, the OGTT can stimulate GH secretion in about 10% of patients with acromegaly.

### IGF-I measurement

The IGF-I level increases in parallel to the log of the GH concentration. It must be determined using age-adjusted norms (levels fall with age). Pregnancy, puberty and the post-pubertal period are accompanied by high IGF-I concentrations. The concentration of IGFBP3, the main IGF carrier protein, is usually increased in patients with acromegaly, but this marker offers little further diagnostic information.

### Stimulation tests

Some patients (up to 50%) have an increase in their GH concentration after thyrotropin-releasing hormone (TRH) and/or gonadotropin-releasing hormone (GnRH) stimulation. These tests have no diagnostic value, however, nor does the response to GHRH.

### Difficult and borderline clinical situations

A few patients with clear clinical signs of acromegaly and a high IGF-I level have a GH nadir of <1 **μ**g/l (3 mIU/l) during OGTT [41]. Thus, some authors have suggested that when chemiluminescence or fluorometric assays with very low detection limits are used (0.10 to 0.30 **μ**g/l, *i.e*. 0.3 to 0.9 mIU/l), the OGTT positivity cutoff should not be 1 **μ**g/l (3 mIU/l) but rather 0.30 **μ**g/l (1 mIU/l) [64]. Forthcoming guidelines should ratify the choice of this new 0.30 **μ**g/l cutoff (1 mIU/l) when using very sensitive GH assays.

Other difficult problems include a typical clinical picture of acromegaly in a patient with normal IGF-I and GH concentrations. This situation probably corresponds to cases in which acromegaly has resolved spontaneously, probably through necrosis of a pituitary adenoma.

In some conditions such as diabetes mellitus, chronic renal failure, pregnancy or at the time of puberty, GH and/or IGF-I measurement cannot be used neither for the diagnosis nor for the assessment of treatment efficacy.

### Differential diagnosis

Lastly, some patients have acromegaloid features with normal GH and IGF-I levels (pseudoacromegaly); in some cases, severe insulin resistance is the proposed mechanism.

## Tumor and functional pituitary assessment

Once the diagnosis has been established, and before initiating treatment for acromegaly, patients must undergo a dual work-up focusing on both the tumor and pituitary function.

### Local tumor effects

#### Headache

Headache is very frequent, and is typically retroorbital or frontal. It is linked to the adenoma and also to GH hypersecretion itself.

#### Visual disorders

**Figure 6:**
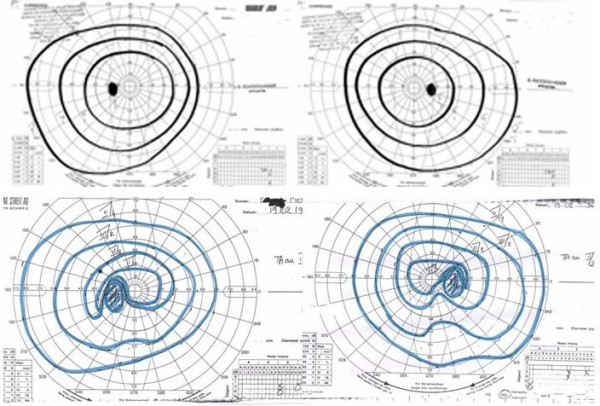
Goldman perimeter showing normal visual fields (upper panel) and bitemporlal quadranopsia due to optic chiasm compression by a pituitary adenoma (lower panel).

When the adenoma grows upwards it can compress the optic chiasm, leading to visual field disturbances, which begin in the mid-periphery of the superior temporal sectors, then progress to bitemporal hemianopsia (Figure 6). Persistent compression can lead to blindness. The visual field and acuity must therefore be routinely assessed.

#### Other manifestations of local tumor effects

In the case of a huge tumor with suprasellar extension into the third ventricle obstructing the foramen of Munro, hydrocephaly can occur. Infrasellar extension can lead to sellar floor lysis and invasion of the sphenoid sinus, with the risk of cerebrospinal fluid rhinorrhea. Lateral extension of the adenoma into the cavernous sinus, which is far more frequent, can compress the III, IV, V, or VI cranial nerves, or even the temporal lobe (with a risk of focal epilepsy).

### Imaging studies

Frontal and profile radiography of the sella turcica should nowdays be abandoned. When formerly performed, they showed an increase in the size of the sella, demineralization of its walls, or local erosion; in patients with very large tumors, the contour of the sella turcica can disappear completely; asymmetrically growing tumors produce a double countour (lateral view) or obliquity of the sellar floor (frontal view).

MRI is tending to replace computed tomography for imaging in this setting. It is currently the neuroradiological examination of choice for all patients with pituitary adenomas, and especially for acromegalic patients.

**Figure 7:**
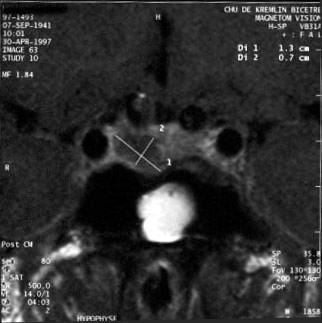
Pituitary microadenoma.

Microadenomas (< 10 mm diameter) appear as rounded, well-circumscribed, homogeneous and discreetly hypointense abnormalities on T1-weighted images (compared with the healthy pituitary or with the white matter of the brain stem); sometimes the adenoma is iso-intense in T1-weighted images and thus difficult to distinguish from normal pituitary before contrast injection. It can be hypo-, iso- or hyperintense on T2-weighted images. After gadolinium injection, microadenomas appear hypointense relative to the rest of the brain parenchyma (Figure 7) and especially to the rest of the pituitary, which shows homogenous uptake. Indirect signs can help, such as asymmetry or bulging of a hemi-pituitary, global bulging with lifting of the sellar diaphragm, and lateral deviation of the pituitary stalk (usually away from the lesion).

**Figure 8:**
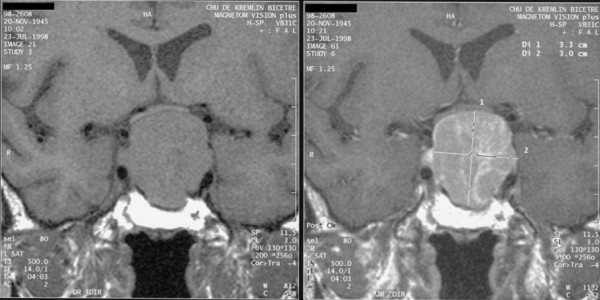
Pituitary macroadenoma with suprasellar extension, compressing the optic chiasm.

Macroadenomas (> 10 mm diameter) are generally iso-intense as compared with the rest of the white matter of brain parenchyma on unenhanced T1-weighted images. Gadolinium contrast medium is intensely taken up by these lesions, which appear hyperintense in comparison with the rest of the white matter of brain parenchyma (Figure 8).

MRI can be used to study possible suprasellar expansion, upwards towards the suprasellar cisterna and the chiasma, which may be compressed, pushed back, or laminated. Extension downwards towards the sphenoid sinus and laterally to the cavernous sinus should also be evaluated. Invasion of the cavernous sinus is difficult to diagnose, as the adenoma may appear to invade the cavernous sinus when it simply abuts the lateral wall. The only indisputable sign of cavernous sinus invasion is when the internal carotid artery appears completely surrounded by the adenoma.

**Figure 9:**
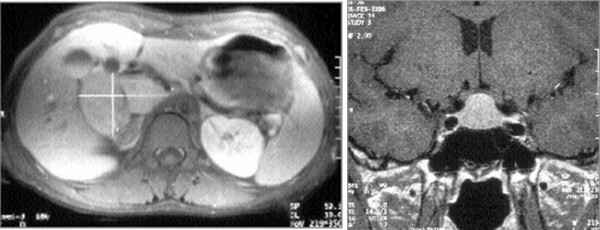
Acromegaly due to ectopic secretion of GHRH by a pancreatic tumor, metastasized to the liver (left) responsible for a pituitary hyperplasia, visible on pituitary MRI (right) and stimulation of normal somatotroph cells, leading to GH hypersecretion and acromegaly.

The lack of clear signs of an adenoma on MRI, or an appearance showing a bulging, hyperplastic pituitary (Figure 9), suggests that the acromegaly is secondary to ectopic GHRH secretion [4].

### Functional pituitary investigations

By developing in the sellar fossa, the adenoma compresses the healthy pituitary (or the pituitary stalk) and can alter physiological pituitary secretion. A gonadotrope deficiency will cause sexual dysfunction and decreased plasma testosterone levels in men, and menstrual disorders in women (amenorrhea in some cases) together with a decline in estradiol levels (without rise in gonadotrophin levels). Thyrotrope deficiency is diagnosed by a decrease in T4 levels, while the TSH level is in the normal range. Corticotrope deficiency can be evaluated by measuring morning plasma cortisol levels and/or testing with metyrapone, corticotropin-releasing hormone (CRH) or ACTH, according to each center habits.

Prolactin (PRL) hypersecretion is present in 30% of cases, either functional (secondary to impairment of hypothalamic production of dopamine or compression of the pituitary stalk by the tumor that impairs dopamine transport to the pituitary), or due to a mixed adenoma secreting both PRL and GH. Free alpha-subunit is hypersecreted in 20% to 40% of cases of acromegaly. Hypersecretion can also affect TSH, both PRL and TSH or, in very rare cases, gonadotrophins or ACTH.

## Management and treatment

### Treatment aims [65]

**Figure 10:**
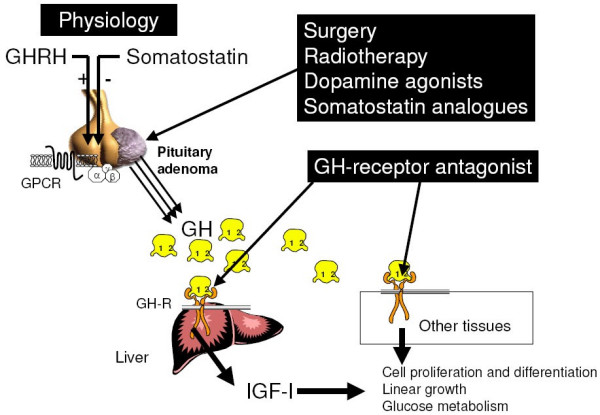
Site of action of the different therapeutic tools in acromegaly. Surgery, radiotherapy, somatostatin anlogues and dopamine agonists act at the level of the pituitary adenoma, while GH-receptor antagonists act in periphery by blocking the GH receptor and thus impairing the effects of GH on the different tissues.

The clinical aims are to relieve symptoms, to reduce the volume of the pituitary tumor, to avoid tumor relapse, and to improve long-term morbidity and mortality. Recent epidemiological studies helped to refine the definitions of "cure" and good disease control, which are now far more precise: the GH concentration (the mean of several samples, or the nadir in the OGTT) must return to less than 2 **μ**g/l or 6 mIU/l (or even 1 **μ**g/l or 3 mIU/l) and the IGF-I level must return to normal [63]. A stepwise therapeutic strategy using surgery and/or radiotherapy and/or medical treatment (Figure 10) allows to achieve these goals.

### Surgery is generally first-line treatment

Tumor excision, usually by the trans-sphenoidal route, is the most rapid way of reducing GH and IGF-I concentrations in patients with acromegaly. Nevertheless, these levels normalize in only 40% to 70% of cases [66-69], depending on the size of the tumor (microadenomas are more amenable to cure), preoperative GH concentrations (the success rate is higher when GH concentrations are low, *i.e*. <10 **μ**g/l or 30 mIU/l), and the surgeon's experience. Endoscopic techniques are now currently used in the majority of expert centers [70,71].

Surgical outcome is carefully assessed at three months. When surgery fails to achieve good disease control, or when surgery is impossible or contraindicated, patients are offered radiotherapy and/or pharmacological treatments.

### Radiotherapy

Radiotherapy in this setting is generally external and centered on the tumor; an average total dose of 50 Gy is delivered in about twenty five daily sessions. Highly focused irradiation (radiosurgery, stereotactic radiotherapy, "gamma-knife", etc.) is now available in some centers, and causes less damage to neighboring tissues. Fractioned irradiation yields GH concentrations below 2 **μ**g/l (6 mIU/l) and normal IGF-I levels in 5% to 60% of patients, depending on the series, after a median follow-up of about 7 years [72-75]. In studies with longer follow-up, fractionated radiotherapy normalizes the IGF-I level in more than 70% of patients after 10 years. Here again, the baseline GH concentration seems to be predictive of treatment outcome.

Radiotherapy leads to variable degrees of anterior pituitary insufficiency in 80% to 100% of patients after 10–15 years. Complications such as radionecrosis and optic neuropathy are now very rare. In contrast, the risk of stroke may be increased, sometimes many years after irradiation [76].

The precise role of stereotactic irradiation in this setting (*e.g*. with a gamma-knife) is becoming clearer [77,78], but long-term studies with strict endpoints are needed to tell whether this approach is as effective and safer than existing alternatives. Results of a French series of 82 patients indicate that efficacy is similar to that of fractionated radiotherapy [79]: an average of 4 years after the procedure, less than 20% of patients have normal IGF-I levels and GH <2 **μ**g/l (<6 mIU/l). In any event, this "radiosurgery" is reserved for patients with small lesions located at least 5 mm from the optic chiasma.

### Medical treatment

#### Dopamine agonists

Bromocriptine attenuates moderately the symptoms of acromegaly and reduces GH concentrations, but it normalizes IGF-I levels in only 10% of patients. Cabergoline appears to be more effective [80,81].

#### Somatostatin analogs

• Mechanism of action. Somatostatin analogs suppress GH secretion by binding to somatostatin receptors, of which there are five subtypes (SST), on somatotrope adenoma cells. These drugs exert their antisecretory and antitumoral effects by acting on SST 2 and 5.

• Available preparations. Octreotide (Sandostatin^®^) can be injected subcutaneously (SC), generally by the patient him/herself, at a dose of 100 to 200 **μ**g two or three times a day. This was the first such analog to be marketed, in the 1980s, and represented a real therapeutic advance [82]. Sustained-release lanreotide (Somatuline^® ^LP 30 mg) was the first slow-release preparation to be marketed. It was injected intramuscularly every 10 to 14 days (the frequency of injections depends on the impact on the GH concentration). Lanreotide is now available for deep SC injection every 28 days, at doses of 60, 90 and 120 mg (Somatuline^® ^Autogel^® ^60, 90 or 120 mg). Octreotide LAR (Sandostatin^® ^LAR 10–20 or 30 mg) is the sustained-release version of octreotide, and is administered intramuscularly, once a month. Treatment is usually started at the median dose, and is then adjusted (decreased or increased) according to the GH concentration. Alternatively, it is possible to increase or decrease the frequency of injections.

• These drugs achieve GH concentrations below 2 **μ**g/l (5 mIU/l) in 60% to 70% of patients, and normalize IGF-1 levels in 50% to 80% of patients [83-90]. Besides their antisecretory effect, somatostatin analogs also reduce the tumor volume (generally the suprasellar portion) in 20% to 70% of patients [88,91]. The reduction in tumor volume is larger when a somatostatin analog is the first-line treatment [88].

• In selected cases (contra-indications to surgery, patients with severe comorbidities who need to be prepared by medical treatment before surgery [92], invasive tumors for which total removal is unlikely [87,93]) somatostatin analogs may be given as first-line therapy.

• Disadvantages: These treatments must be continued indefinitely because Somatostatin analog only suppress GH hypersecretion. They have gastrointestinal adverse effects, which are generally transient, and cause gallstones in 10% to 20% of patients. They are also expensive.

#### The GH receptor antagonist, pegvisomant (Somavert^®^)

Pegvisomant has a different mechanism of action. It acts in the periphery, blocking the effects of GH on its target organs by binding to GH receptors and preventing their dimerization; this blocks GH signal transduction and inhibits GH activity, including IGF-I production [94]. As pegvisomant inhibits the action of GH but not its secretion, GH concentrations cannot be used to evaluate treatment efficacy. IGF-I is used as a surrogate marker, together with clinical parameters. Pegvisomant is administered subcutaneously at a daily dose of 10, 15 or 20 mg (sometimes more), the dose being adapted to the hormone response (IGF-I normalization). Pegvisomant is highly effective, as IGF-I levels normalize in more than 90% of patients [95,96]. For the moment this treatment is reserved for patients in whom somatostatin analogs fail. A small increase in tumor volume is observed in a few patients (possibly related to the natural history of the adenoma, or to growth stimulation), and this may justify combination with an somatostatin to reduce tumor volume [97]. Tumor volume must therefore be monitored (by MRI) during this treatment. Available clinical data on pegvisomant concern a relatively small number of patients and relatively short treatment periods. The adverse effects are limited to rare cases of increased transaminases that generally normalize either after interruption of the treatment or spontaneously. Exceptional cases of true hepatitis were reported [98].

### Current therapeutic strategy

The advantages, disadvantages and costs of treatment must be taken into account.

**Figure 11:**
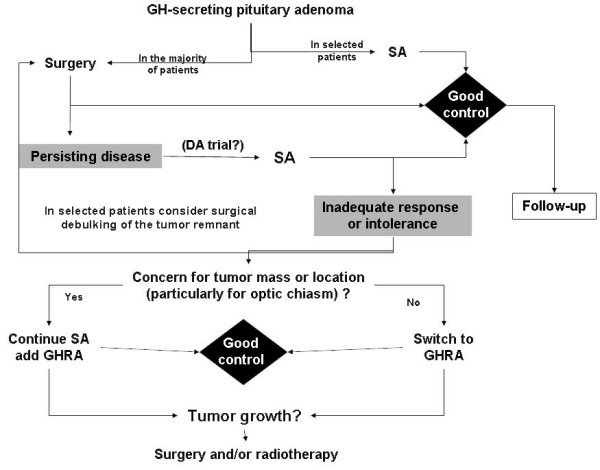
Strategy proposed by the Authors for the current management of acromegaly. SA: somatostatin analogs; DA: dopamine agonists, GHRA: GH-receptor antagonist (pegvisomant).

A therapeutic strategy is proposed by the Authors on Figure 11. Currently, if surgical treatment fails to cure acromegaly, medical treatment with somatostatin analogs is recommended rather than radiotherapy. If somatostatin analog therapy fails, it may be interesting to propose a reoperation in case of important tumor remnant, before another trial of somatostatin analog. Otherwise, one can propose pegvisomant before resorting to radiotherapy. The cost of these medical treatments, which may be required indefinitely, must be weighed up against the risks of radiotherapy. In any event, medical treatment will be necessary while waiting for the effects of radiotherapy to appear.

If surgery and radiotherapy are contraindicated, first-line somatostatin therapy may be proposed.

All these treatments must be re-assessed on a yearly basis.

## Prognosis and outcome

Acromegaly is associated with increased mortality [1]. According to series published in the 1980–1990s, about 60% of patients die from cardiovascular disease, 25% from respiratory complications, and 15% from cancer. If left untreated, patients with acromegaly would die about 10 years earlier than healthy subjects. Several studies have shown that cerebrovascular disorders are a frequent cause of death, especially among women, but they involved patients treated in various ways and many years ago (craniotomy, radiotherapy), and a deleterious effect of these treatments (especially radiotherapy) cannot be ruled out [99,100]. The standardized mortality index (the ratio of observed mortality in the acromegalic population to expected mortality in the general population) ranges from 1.2 to 3.3. The post-treatment GH concentration is probably the best predictor of survival, for all causes of death, independently of the types of complication. Thus, life expectancy outcomes can be stratified according to the post-treatment GH concentration: if GH secretion is controlled (<2 **μ**g/l, or <5 mIU/l, or IGF-I normalization), life expectancy merges with that of the matched general population [99,101]. High GH/IGF-I concentrations, arterial hypertension, and cardiomyopathy are factors in a poor prognosis, while the duration of symptoms and other factors (diabetes, lipid disorders and cancer) are less important [30]. Quality of life is also altered in acromegaly, and is partially improved by effective treatment [102]. Finally, it must be stressed that, with the current therapeutic strategy, the vast majority of acromegalic patients have very good control of GH/IGF-I secretion and no problems relating to tumor growth. Adverse effects are infrequent and minor, even in the very long term; this picture is very different from the situation only 20 years ago, before the somatostatin analogs era. In addition, the use of more stringent criteria to define cure, together with aggressive treatment of comorbidity, has significantly improved the outlook for patients with acromegaly [103]. However, even if patients are cured or well-controlled, sequelae (joint pain, deformities and altered quality of life) often remain.

## Abbreviations

ACTH: corticotrophin; AIP: aryl hydrocarbon receptor interacting protein; CG: chorionic gonadotropin hormone; CRH: corticotropin-releasing hormone; ECG: electrocardiography; ELISA: enzyme-linked immunoabsorbent assay; FSH: follicle-stimulating hormone; GH: growth hormone; GH-BP: GH-binding protein; GHRH: growth hormone-releasing hormone; GnRH: gonadotropin-releasing hormone; ICMA: immunochemiluminescent assay; IGF-I: insulin-like growth factor I; IRMA: immunoradioimmunometric assay; IS: International Standard; LH: luteinizing hormone; MEN1: multiple endocrine neoplasia type 1; MRI: magnetic resonace imaging; OGTT: oral glucose tolerance test; PRL: prolactin; PRKAR1A: 1-a subunit of protein kinase A; PTTG: pituitary tumor transforming gene; RIA: radio-immunoassay; TRH: thyrotropin releasing hormone; TSH: thyroid-stimulating hormone; SST: somatostatin receptor subtypes

## Competing interests

The Service d'Endocrinologie et des Maladies de la Reproduction, Université Paris-Sud 11, receives unrestricted educational and research grants from Novartis, Ipsen and Pfizer. PC received consulting and lecture fees from Novartis, Ipsen and Pfizer. SS has nothing to declare.

## Authors' contributions

The two authors equally contributed to this review article. They read and approved the final version of the manuscript.

## Consent

Written consent for publication of photographs was obtained from the patients.
